# Draft Genome Sequence of *Vibrio* sp. Strain Vb278, an Antagonistic Bacterium Isolated from the Marine Sponge *Sarcotragus spinosulus*

**DOI:** 10.1128/genomeA.00521-15

**Published:** 2015-05-28

**Authors:** Ana C. S. Gonçalves, Telma Franco, Gianmaria Califano, Scot E. Dowd, Georg Pohnert, Rodrigo Costa

**Affiliations:** a Department of Biological Sciences, State University of Santa Cruz, UESC, Ilhéus, Bahia, Brazil; b Centre of Marine Sciences (CCMar), Microbial Ecology and Evolution Research Group, University of Algarve (UAlg), Gambelas, Faro, Portugal; c MR DNA, Molecular Research LP, Shallowater, Texas, USA; d Institute for Inorganic and Analytical Chemistry (IAAC), Friedrich Schiller University Jena, Jena, Germany

## Abstract

We report here the draft genome sequence of *Vibrio* sp. Vb278, a biofilm-producing strain isolated from the marine sponge *Sarcotragus spinosulus*, showing *in vitro* antibacterial activity. The annotated genome displays a range of symbiotic factors and the potential for the biosynthesis of several biologically active natural products.

## GENOME ANNOUNCEMENT

*Vibrio* species are known for their metabolic and genotypic plasticity, having widespread occurrence in marine and estuarine ecosystems as members of either planktonic (1, 2) or symbiotic communities (3, 4). To date, much research has been performed, e.g., on *Vibrio*-squid symbiosis, and a mechanistic view of this particular interaction has been achieved (5). Yet, the precise roles of *Vibrio* spp. across several foundational marine symbiotic consortia, such as coral, sponge, and seaweed holobionts, where they emerge as one of the most profuse cultivatable members (4, 6, 7), remain far from being fully understood. To gain better insight into the antimicrobial potential and life strategies of symbiotic *Vibrio* species, we sequenced the genome of *Vibrio* sp. strain Vb278, a biofilm-forming bacterium displaying *in vitro* antagonism toward *Escherichia coli* and *Staphylococcus aureus* (4).

Strain Vb278 was isolated from the marine sponge *Sarcotragus spinosulus* using marine agar medium. The animal host was sampled at 15 m depth off the coast of Galé Alta (37°04′09.6N 8°19′52.1 W), Algarve, southern Portugal (4). Genomic DNA of strain Vb278 was extracted from a pelletized, freshly grown culture (48 h of incubation at 23°C) using the Wizard genomic DNA purification kit (Promega Corporation, Madison, WI) and sequenced on an Illumina MiSeq platform at MR DNA (Shallowater, TX). The sequence output was 1.22 Gb, comprising 2 × 301-bp paired-end reads, allowing for an estimated 228× coverage of the targeted genome. The obtained sequence reads were *de novo* assembled by MR DNA into 14 contigs (longest, 1,197,025 bp) with the NGen DNA assembly software by DNAStar, Inc. The resulting draft genome sequence was annotated using the Rapid Annotation using Subsystems Technology (RAST) prokaryotic genome annotation server, version 2.0 (8). The genome is 5,359,740 bp long and has a G+C content of 44.6%. In total, 4,709 coding sequences (CDSs) were identified, in addition to 114 tRNAs and 37 rRNAs.

*Vibrio* sp. Vb278 presents 99.7% and 99.3% 16S rRNA gene similarity with type strains *Vibrio gigantis* LGP 13 (9) and *Vibrio crassostreae* LG 7 (10), respectively, both isolated from cultured oysters (*Crassostrea gigas*). Its genome displays a vast repertoire of carbohydrate-degrading genes, along with the complete vibrioferrin (siderophore) biosynthesis and transport gene clusters, suggesting that strain Vb278 possesses excellent nutrient-scavenging capacities. Of note is the presence of 14 of the 18 members of the symbiotic colonization and sigma-dependent biofilm formation (polysaccharide biosynthesis) gene cluster Syp, originally described for *Aliivibrio fischeri* (formerly *Vibrio fischeri*) (11). We further identified autoinducer 2 (AI-2) production (LuxS), binding (LuxP), and sensor kinase (LuxQ) protein biosynthesis genes, type I, II IV, and VI secretion system-encoding genes, and the full bacteriocin production operon. Altogether, the genome of *Vibrio* sp. strain Vb278 features varied adaptive strategies for symbiotic living, underlying the strain's high virulence potential and host colonization aptitude.

### Nucleotide sequence accession numbers.

The genome sequence of *Vibrio* sp. strain Vb278 has been deposited in the DDBJ/ENA-EBI/NCBI databases under the accession no. CVNE00000000. The version described in this study is CVNE00000000.1 and consists of contig sequences CVNE01000001 to CVNE01000014.
